# Hsa_circ_0017639 regulates cisplatin resistance and tumor growth via acting as a miR-1296-5p molecular sponge and modulating sine oculis homeobox 1 expression in non-small cell lung cancer

**DOI:** 10.1080/21655979.2022.2053810

**Published:** 2022-03-28

**Authors:** Feiyun Chang, Jiali Li, Quan Sun, Shuqing Wei, Yongming Song

**Affiliations:** Department of Thoracic Surgery, Shanxi Province Cancer Hospital, Shanxi Hospital Affiliated to Cancer Hospital, Chinese Academy of Medical Sciences, Cancer Hospital Affiliated to Shanxi Medical University, Taiyuan, Shanxi, China

**Keywords:** DDP, IC_50_, NSCLC, circ_0017639, miR-1296-5p, SIX1

## Abstract

Cisplatin (DDP)-induced chemoresistance is an important reason for the failure of non-small cell lung cancer (NSCLC) treatment. Circular RNAs (circRNAs) participate in the chemoresistance of diverse cancers. However, the function of hsa_circ_0017639 (circ_0017639) in the DDP resistance of NSCLC is unclear. Forty-one NSCLC samples (21 DDP-resistant samples and 20 DDP-sensitive samples) were utilized in the research. The relative expression levels of some genes were determined by real-time quantitative polymerase chain reaction (RT-qPCR). 3-(4,5-Dimethylthiazol-2-yl)-2,5-Diphenyltetrazolium Bromide (MTT) assay for half-maximal inhibitory concentration (IC_50_) value of DDP and cell viability, colony formation and 5-ethynyl-2’-deoxyuridine (EDU) assays for cell proliferation, flow cytometry assay for cell apoptosis, transwell assay for cell invasion and wound-healing assay for cell migration were performed. The regulation mechanism of circ_0017639 was demonstrated by a dual-luciferase reporter assay. We observed higher levels of circ_0017639 in DDP-resistant NSCLC samples and cells. Functionally, circ_0017639 silencing decreased tumor growth and elevated DDP sensitivity *in vivo* and induced apoptosis, repressed proliferation, invasion, and migration of DDP-resistant NSCLC cells *in vitro*. Mechanically, circ_0017639 modulated sine oculis homeobox 1 (SIX1) expression via sponging microRNA (miR)-1296-5p. Also, miR-1296-5p inhibitor restored circ_0017639 knockdown-mediated impacts on cell DDP resistance in DDP-resistant NSCLCs. Furthermore, SIX1 overexpression counteracted the inhibiting impact of miR-1296-5p upregulation on DDP resistance and malignant phenotypes of DDP-resistant NSCLC cells. In conclusion, circ_0017639 conferred DDP resistance and promoted tumor growth via elevating SIX1 expression through sequestering miR-1296-5p in NSCLC, providing a new mechanism for understanding the chemoresistance and progression of NSCLC.

## graphical abstract

**Figure:**
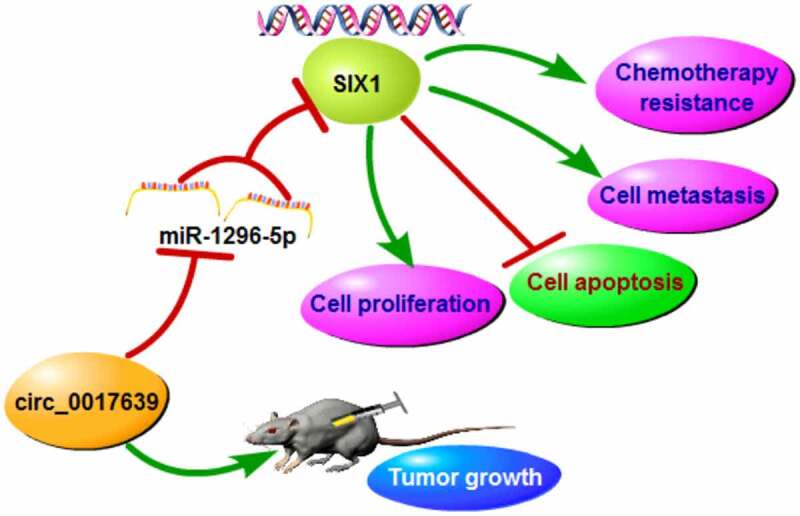

## Introduction

Lung cancer causes a higher mortality rate than any other malignant tumor [1]. Despite the remarkable development of immunotherapy and molecular-targeted therapy, the 5-year overall survival rate of lung cancer patients is still very low [2,3]. Non-small cell lung cancer (NSCLC) is the major subtype of lung cancer (approximately 85% of lung cancer cases) [4,5]. Cisplatin (DDP) is a first-line chemotherapeutic drug for advanced NSCLC. Normally, patients with NSCLC respond to the initial treatment of DDP, but resistance often develops, resulting in poor survival [6].

Recently, changes in circular RNAs (circRNAs) levels have been observed in DDP-resistant NSCLC cells [7]. CircRNAs are mainly produced through the process of back-splicing, in which the 3’ and 5’ends of the precursor RNA are cleaved and joined by the splicing mechanism [8]. Moreover, circRNAs have the advantages of specific expression in different cell types, tissue types, and developmental stages [9]. Researchers uncovered that circRNAs participate in miscellaneous pathological processes, including tumorigenesis, tumor metastasis, and drug resistance [10]. For instance, circRNA circ-fibroblast growth factor receptor 1 accelerated anti-programmed death-1 resistance and tumor growth through upregulating C-X-C motif chemokine receptor 4 in NSCLC [11]. Another study exposed that decreased circRNA circ_0001946 expression in NSCLC was related to DDP insensitivity [12]. By analyzing the GSE112214 microarray, we found that circRNA hsa_circ_0017639 (circ_0017639) is a differentially expressed circRNA in NSCLC tissues. Li and colleagues revealed that circ_0017639 plays an oncogenic function in gastric cancer [13]. At present, the action of circ_0017639 in NSCLC is indistinct, so it aroused our interest.

Researchers have demonstrated that circRNAs exert a crucial regulatory action as microRNA (miR) decoys/sponges in tumors [14]. MiRs are important post-transcriptional regulators of gene expression, and they control many cellular processes and developmental in eukaryotic organisms [15]. MiR-1296-5p exerts a tumor-suppressing impact in osteosarcoma [16], breast cancer [17], and hepatocellular cancer [18]. However, the action of miR-1296-5p in NSCLC resistance to DDP has not been verified. Transcription factor sine oculis homeobox 1 (SIX1) is a member of the sine oculis (So/Six) homeobox protein family and a powerful regulator of organogenesis [19]. SIX1 is considered to be a cancer fetoprotein, and its inappropriate re-expression can lead to genome instability, malignant transformation, and metastasis [20]. Moreover, SIX1 participates in the chemoresistance of many cancers [^21–23^]. Upregulation of SIX1 can confer paclitaxel resistance in breast cancer [24], DDP resistance in NSCLC [25], and 5-fluorouracil resistance in hepatocellular cancer [26]. However, the mechanism related to SIX1 dysregulation in DDP resistance of NSCLC remains unclear.

Thus, the research was to explore the function of circ_0017639 in NSCLC resistance to DDP and its regulatory mechanism. Herein, circ_0017639 was verified as an adverse circRNA in NSCLC. Mechanically, circ_0017639 upregulation endowed DDP resistance in NSCLC by elevating the SIX1 expression via serving as a miR-1296-5p decoy.

## Materials and methods

### Biological specimens

Forty-one human NSCLC samples and matched adjacent normal samples were collected under the authorization of the Ethical Committee of Shanxi Province Cancer Hospital, Shanxi Hospital Affiliated to Cancer Hospital, Chinese Academy of Medical Sciences, Cancer Hospital Affiliated to Shanxi Medical University. According to the DDP treatment response, 41 NSCLC samples were divided into two parts: DDP-resistant NSCLC group (21 samples) and DDP-sensitive NSCLC group (20 samples). All registered NSCLC patients provided informed consent and underwent surgical resection at Shanxi Province Cancer Hospital, Shanxi Hospital Affiliated to Cancer Hospital, Chinese Academy of Medical Sciences, Cancer Hospital Affiliated to Shanxi Medical University.

### Cell culture

Human bronchial epithelial cells (HBE), HEK-293 T cells, and lung cancer cells (H460, H1650, A549, and H1299) (Procell, Wuhan, China) were maintained under the right conditions (5% carbon dioxide and 37°C). HEK-293 T and A549 cells were, respectively, cultured in DMEM (PM150210, Procell) and Ham’s F-12 K (PM150910, Procell), whereas other cells were cultured in Roswell Park Memorial Institute-1640 Medium (PM150110, Procell). All media utilized for cell growth were supplemented with 10% Fetal Bovine Serum (FBS) (164,210–500, Procell) and 1% Penicillin/Streptomycin (PB180120, Procell).

### Establishment of DDP-resistant NSCLC cells

DDP-resistant NSCLC cells (A549/DDP and H1299/DDP) were established by a gradual increase in DDP concentration as described previously [27]. Briefly, A549 cells were initially exposed to DDP (0.5 μg/mL) DDP (Sigma, St Louis, MO, USA) for 3 days and then recovered within 3 days. After three cycles, the concentration of DDP was gradually increased to 1, 2, 4, 8 and 10 μg/mL to generate DDP-resistant NSCLC cells.

### Oligonucleotides and plasmids

Small interference (si) RNA against circ_0017639 (si-circ_0017639), miR-1296-5p inhibitor (anti-miR-1296-5p), miR-1296-5p mimic (miR-1296-5p), as well as the negative controls of oligonucleotide sequences mentioned above (si-NC, anti-miR-NC, and miR-NC) were synthesized by AoKe Biotech (Beijing, China). For pCD5-ciR-circ_0017639 (circ_0017639) and pcDNA-SIX1 (SIX1) plasmids, the cDNA sequences of circ_0017639 and SIX1 were inserted into empty pCD5-ciR (vector) (Geneseed, Guangzhou, China) and pcDNA (Thermo Fisher, Burlington, ON, Canada) vectors, respectively. Transfection of DDP-resistant NSCLC cells with specific oligonucleotides and/or plasmids was performed using Lipofectamine 3000 (Thermo Fisher) or Lipofectamine RNAiMax (Thermo Fisher).

### RNA isolation and RNase R digestion

Total RNA was isolated using an RNA Isolation Kit (Qiagen, Hilden, Germany) following the manufacturer’s steps. RNA from the nucleus or cytoplasm was extracted using the Cytoplasmic & Nuclear RNA Purification Kit (Norgen Biotek, Thorold, Canada) based on the manufacturer’s procedures. To validate the circular structure of circ_0017639, total RNA from DDP-resistant NSCLC cells was incubated with diethylpyrocarbonate (DEPC)-treated water (Sigma) or RNase R (20 U/μL, Sigma) [28].

### Real-time quantitative polymerase chain reaction (RT-qPCR)

The synthesis of complementary DNA from total RNA was conducted with Prime Script™ RT reagent kit (TaKaRa, Dalian, China) or miScript II RT Kit (Qiagen). Quantitative PCR was set up with the SYBR Green kit (Qiagen) in triplicate, and data were calculated using the 2^−ΔΔCt^ method [29]. Primer sequences are exhibited in Table 1. U6 or β-Actin was used as an endogenous reference.

**Table 1.** Primer sequences used for RT-qPCR

Genes	Primer sequences (5’-3’)
circ_0017639	Forward (F): 5’-CAGATTTGCGAAGCCATTTC-3’
	Reverse (R): 5’-TCCTCGAACCAGTCAAGTCA-3’
SIX1	F: 5’-AGGTCAGCAACTGGTTTAAGAACC-3’
	R: 5’-GAGGAGAGAGTTGGTTCTGCTTG-3’
β-Actin	F: 5’-CAGCCATGTACGTTGCTATCCA-3’
	R: 5’-TCACCGGAGTCCATCACGAT-3’
miR-1296-5p	F: 5’-CGTTAGGGCCCTGGCTCC-3’
	R: 5’-CAGTGCGTGTCGTGGAGT-3’
U6	F: 5’-CTCGCTTCGGCAGCACA-3’
	R: 5’-AACGCTTCACGAATTTGCGT-3’

### 3-(4,5-Dimethylthiazol-2-yl)-2,5-Diphenyltetrazolium Bromide (MTT) assay

The half-maximal inhibitory concentration (IC_50_) value and viability of DDP-resistant NSCLC cells were evaluated using the MTT assay following the previous description [30]. In brief, cells with or without DDP treatment were incubated for 48 h, followed by co-incubating with the MTT solution (10 μL) (Roche, Basel, Switzerland) for 4 h. Following this, the purple crystals were dissolved, and the optical density was analyzed using an absorbance reader (BioTek, Winooski, VT, USA).

### Colony formation assay

A previous report was referenced to perform this experiment [31]. Approximately 2 × 10^2^ DDP-resistant NSCLC cells were seeded into 6-well plates. After culture for 12 days, the cloned cells were stained with 0.1% crystal violet (Beyotime, Shanghai, China) and then photographed and counted using a microscope (Olympus, Tokyo, Japan).

### 5-ethynyl-2’-deoxyuridine (EDU) assay

The Cell-Light™ EDU Apollo 567 In Vitro Imaging Kit (Thermo Fisher) was utilized to observe the EDU-positive cells following the manufacturer’s instructions. Nuclei were labeled with blue fluorescence using 4,6-diamidino-2-phenylindole (DAPI). Images were captured using a microscope (Olympus).

### Flow cytometry assay

DDP-resistant NSCLC cells were harvested and then trypsinized gently. Following this, the cells were re-suspended with 1 × binding buffer and then stained with Annexin V-fluorescein isothiocyanate (FITC) (5 μL) and phosphatidylinositol (PI) (5 μL) using the Annexin V-FITC/PI apoptosis detection kit (Becton Dickinson, Santa Cruz, California, USA). After incubation for 5 min on ice, the cells were analyzed using a FACS Verse flow cytometer (Becton Dickinson).

### Transwell invasion assay

Cell invasion was analyzed using transwell chambers with Matrigel (Costar, Cambridge, MA, USA) as previously depicted [31]. Approximately 1 × 10^5^ DDP-resistant NSCLC cells in 200 μL serum-free medium were seeded into the upper compartment, and 600 μL completed media containing 10% FBS (Procell) was added to the lower compartment. 24 h later, the invaded cells were stained with 0.1% crystal violet (Beyotime), followed by counting using a microscope (Olympus) in 5 random fields.

### Wound-healing assay

The wound-healing assay was utilized for migration analysis [32]. In short, about 1 × 10^3^ DDP-resistant NSCLC cells were seeded into 6-well plates. Wounds were scratched in the cell monolayer using a pipette tip when the cells reach 90–100% confluence. After rinsing with phosphate-buffered saline, the cells were incubated for 24 h under the right conditions. A microscope (Olympus) was utilized to capture the pictures at 0 and 24 h.

### Dual-luciferase reporter assay

The fragments of wild type (WT) circ_0017639 and WT SIX1 3’UTR and their mutant (MUT) sequences were ligated into the pMIR-REPORT vector (Thermo Fisher). Luciferase assay was performed by co-transfecting with the luciferase vector along with miR-1296-5p or miR-NC and pRL-TK plasmids (Thermo Fisher) into DDP-resistant NSCLC cells. Analysis of luciferase activity was done with the Pierce™ Renilla-Firefly Luciferase Dual Assay Kit (Thermo Fisher).

### Western blotting

Total protein was extracted using the RIPA buffer (Beyotime). Western blotting was done as described by Chen *et al.* [33] with specific primary antibodies against SIX1 (ab243247, Abcam, USA), matrix metalloproteinase 9 (MMP9) (ab58803, Abcam), and Cleaved-caspase-3 (Cleaved-caspase-3, Abcam), and β-Actin (ab8226, Abcam). Development of the blots was done with the SuperSignal West Pico PLUS Chemiluminescent Substrate (Thermo Fisher).

### In vivo *experiments*

The animal assay was manipulated under the authorization of the Animal Care Committee of Shanxi Province Cancer Hospital, Shanxi Hospital Affiliated to Cancer Hospital, Chinese Academy of Medical Sciences, Cancer Hospital Affiliated to Shanxi Medical University. For xenograft assay, 32 BALB/c nude mice (Vital River Laboratory, Beijing, China) were randomly divided into 4 groups (8 mice in each group) and then processed as follows: (1) mice were injected with sh-NC-transduced H1299/DDP cells (4 × 10^6^) and administrated with phosphate-buffered saline or DDP; (2) mice were injected with sh-circ_0017639-transduced H1299/DDP cells (4 × 10^6^) and administrated with phosphate-buffered saline or DDP. For DDP treatment, mice were intraperitoneally administrated with DDP (5 mg/kg) every 3 days from day 8. Tumor volume was measured every 3 days (Volume = (length × width^2^)/2) from the beginning of DDP administration. Xenograft tumors were excised, weighed, and paraffin-embedded after injection for 23 days. Paraffin-embedded tissues were cut (5.0 μm sections) and subjected to immunohistochemistry (IHC) to detect the level of SIX1 as described previously [34]. An antibody against SIX1 (PA5-51,654, 1:500, Thermo Fisher) was used for IHC analysis.

### Statistical analysis

At least three biological repeats were performed for each experiment. Data were analyzed using GraphPad Prism 8 software (GraphPad, La Jolla, CA, USA) and presented as mean ± standard deviation. Correlation was evaluated using Pearson’s correlation analysis. Significance was determined using the Student’s *t*-test or analysis of variance. If the *P*-value was below 0.05, the results were considered statistically significant.

## Results

The current research aimed to investigate the action of circ_0017639 in the resistance of NSCLC to DDP. Our findings demonstrated that circ_0017639 promoted DDP resistance and tumor progression via sequestering miR-1296-5p and subsequent elevating SIX1 expression. This study provided new insights into DDP resistance and highlighted a potential target for improving DDP therapy in NSCLC.

### Identification of circ_0017639 expression in DDP-resistant and DDP-sensitive NSCLC samples

**Figure 1.**
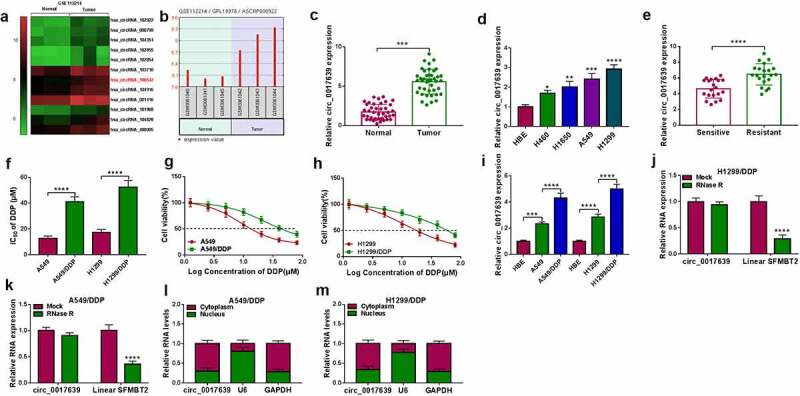
**Circ_0017639 was overexpressed in DDP-resistant NSCLC samples and cells**. (a) Heatmap exhibited 12 significantly upregulated circRNAs in NSCLC samples with respect to matching adjacent normal samples (GSE112214 microarray). (b) A diagram exhibited the level of circ_0017639 in NSCLC samples and 3 matching adjacent normal samples (GSE112214). (c and d) RT-qPCR analysis of circ_0017639 in 41 NSCLC samples (with respect to adjacent normal samples) and 4 NSCLC cell lines (with respect to HBE cells). (e) RT-qPCR analysis of circ_0017639 in 21 DDP-resistant NSCLC samples and 20 NSCLC DDP-sensitive NSCLC samples. (f-h) Analysis of the IC_50_ value of DDP-resistant NSCLC cells and their parental cells by MTT assay. (i) Assessment of circ_0017639 in NSCLC cells (compared with HBE cells) and DDP-resistant NSCLC cells (compared with their parental cells) by RT-qPCR. (j and k) The circular structure of circ_0017639 in DDP-resistant NSCLC cells was verified by RNase R treatment and RT-qPCR analysis. (l and m) Subcellular fractionation assay was carried out to analyze the cellular distribution of circ_0017639 in DDP-resistant NSCLC cells. *P < 0.05, **P < 0.01, ***P < 0.001, and ****P < 0.0001.

To identify the potential circRNAs associated with the DDP resistance of NSCLC, we analyzed the GSE112214 microarray. Heatmap showed the 12 observably upregulated circRNAs in GSE112214 (*P* < 0.001) (Figure 1(a)). The difference of has_circRNA_100542 (circ_0017639) was more obvious than other circRNAs and it had been reported to play an oncogenic role in gastric cancer [13]. Therefore, we speculated that circ_0017639 might be involved in the regulation of lung cancer progression. A diagram showed the level of circ_0017639 in 3 NSCLC samples and 3 matching adjacent normal samples in the GSE112214 microarray (Figure 1(b)). We also observed that circ_0017639 was overexpressed in 41 NSCLC samples (with respect to adjacent normal samples) and 4 NSCLC cell lines (with respect to HBE cells) (Figure 1(c-d)). Notably, circ_0017639 expression was significantly elevated in 21 DDP-resistant samples compared to 20 DDP-sensitive samples (Figure 1(e)). We then constructed DDP-resistant NSCLC cell lines and their IC_50_ values were about 3 times greater than that of their parental cells (Figure 1(f-h)). There was an apparent elevation in circ_0017639 expression in DDP-resistant cells respective to their parental cells (Figure 1(i)). As exhibited in Figure 1(j)–1 K, circ_0017639 was resistant to RNase R treatment in contrast to linear scm like with four mbt domains 2 (SFMBT2). Subcellular fractionation assay showed that the cellular distribution of circ_0017639 was mainly distributed in the cytoplasm of DDP-resistant NSCLC cells, suggesting that circ_0017639 might function as a miRNA molecular sponge (Figure 1l–1 M). Overall, these results manifested that higher levels of circ_0017639 might be related to the DPP resistance of NSCLC.

### Circ_0017639 silencing reduced the resistance and malignancy of DDP-resistant NSCLC cells

**Figure 2.**
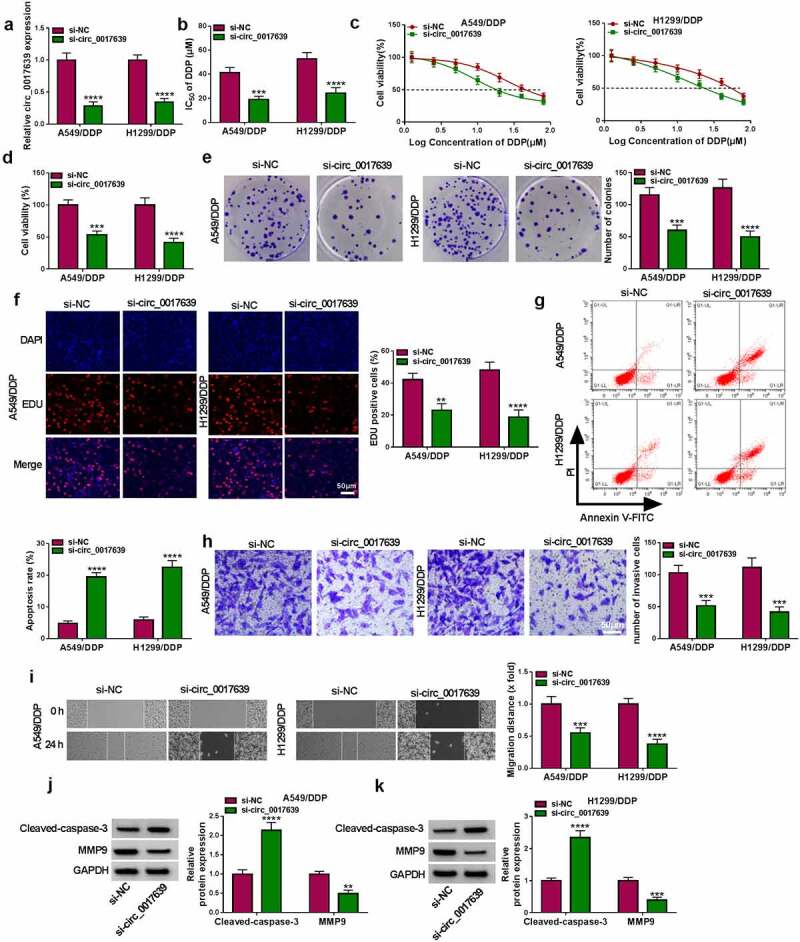
**Influence of circ_0017639 inhibition on the sensitivity and malignancy of DDP-resistant NSCLC cells**. (a) RT-qPCR analysis of circ_0017639 in DDP-resistant NSCLC cells transfected with si-circ_0017639 or si-NC. (b and c) MTT assay assessed the IC_50_ value and viability of circ_0017639-inhibiting DDP-resistant NSCLC cells and control cells. (d-h) The colony formation, proliferation, apoptosis, invasion, and migration of circ_0017639-inhibiting DDP-resistant NSCLC cells and control cells were analyzed by colony formation, EDU, flow cytometry, transwell invasion, and wound-healing assays. (i and j) Western blotting detected MMP9 and Cleaved-caspase-3 protein levels in DDP-resistant NSCLC cells transfected with si-circ_0017639 or si-NC. **P < 0.01, ***P < 0.001, and ****P < 0.0001.

To test the potential role of circ_0017639 in the DDP resistance of NSCLC, we transfected si-circ_0017639 into DDP-resistant NSCLC cells to silence circ_0017639 expression. The results exhibited a lower level in circ_0017639 expression after si-circ_0017639 transfection than the control group (Figure 2(a)). Moreover, circ_0017639 inhibition resulted in a marked decrease in the IC_50_ value and viability of DDP-resistant cells (Figure 2(b-c)). We also observed that the number of colonies and EDU-positive cells was less in the circ_0017639-inhibiting DDP-resistant cells (Figure 2(d,e)). Also, the apoptotic rate of circ_0017639-inhibiting DDP-resistant NSCLC cells was elevated (Figure 2(f)). Furthermore, the invasion and migration abilities of DDP-resistant cells were repressed after circ_0017639 knockdown (Figure 2(g-h)). In addition, circ_0017639 inhibition decreased the MMP9 protein level, whereas elevated the Cleaved-caspase-3 protein level in DDP-resistant NSCLC cells (Figure 2(i-j)). Collectively, these results manifested that circ_0017639 inhibition decreased the resistance and malignancy of DDP-resistant NSCLC cells.

### Circ_0017639 acted as a miR-1296-5p sponge

**Figure 3.**
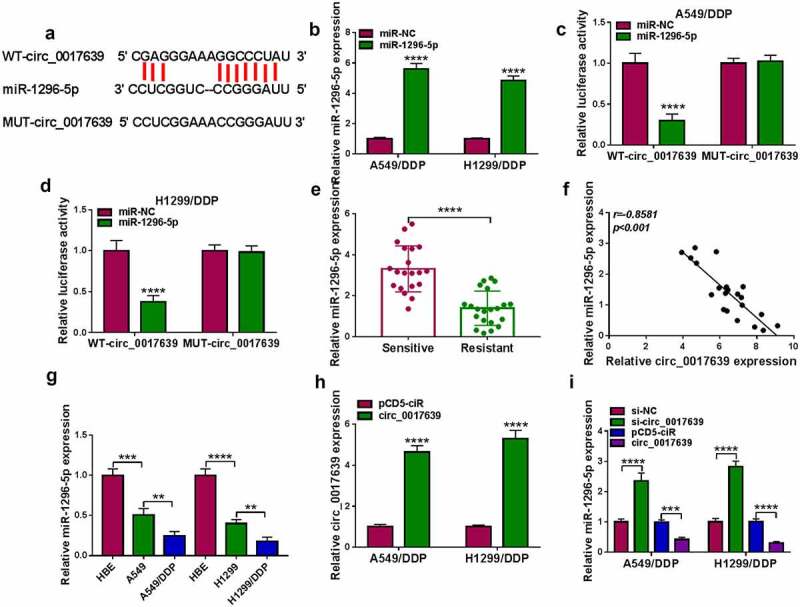
**Identification of circ_0017639 as a miR-1296-5p sponge**. (a) Schematic illustrating the sequence alignment of circ_0017639 with miR-1296-5p. (b) Analysis of miR-1296-5p in DDP-resistant NSCLC cells with miR-1296-5p mimic or miR-NC. (c and d) Dual-luciferase reporter assay analysis of the luciferase activities of WT-circ_0017639 and MUT-circ_0017639 plasmids in the presence of miR-1296-5p mimic or miR-NC in DDP-resistant NSCLC cells. (e) Expression of miR-1296-5p in 21 DDP-resistant NSCLC samples and 20 NSCLC DDP-sensitive NSCLC samples was analyzed by RT-qPCR. (f) Pearson’s correlation analysis uncovered the correlation between miR-1296-5p and circ_0017639 in DDP-resistant NSCLC samples. (g) RT-qPCR analysis of miR-1296-5p in DDP-resistant NSCLC cells (in contrast to their parent cells) and NSCLC cells (in contrast to HBE cells). (h) RT-qPCR analysis of circ_0017639 in DDP-resistant NSCLC cells with circ_0017639 or pCD5-ciR. (i) Effects of circ_0017639 inhibition and overexpression on miR-1296-5p expression in DDP-resistant NSCLC cells. **P < 0.01, ***P < 0.001, and ****P < 0.0001.

It has been proved that circRNAs can exert functions via sponging miRs [35]. Bioinformatics analysis (circinteractome) predicted that there were many miRNAs that might bind to circ_0017639. Through literature review, seven miRNAs (miR-1208 [36], miR-1305 [37], miR-188-3p [38], miR-198-5p [39], miR-224-5p [40], miR-885-3p [41] and miR-1296-5p [42]) that are lowly expressed in lung cancer and inhibit the progression of lung cancer were selected for further analysis. Also, silenced circ_0017639 expression markedly increased the expression levels of miR-1305, miR-224-5p, miR-885-3p and miR-1296-5p, and miR-1296-5p with the largest differential change was selected as the candidate miRNA (supplementary Fig. 1A). The binding sites between miR-1296-5p and circ_0017639 are provided in Figure 3(a). We then performed a dual-luciferase reporter assay to examine whether miR-1296-5p target circ_0017639. The overexpression efficiency of miR-1296-5p mimic is displayed in Figure 3(b). Also, miR-1296-5p mimic repressed the luciferase activity of WT-circ_0017639 in DDP-resistant NSCLC cells, but the luciferase activity of MUT-circ_0017639 was not affected (Figure 3(c-d)). We also observed lower levels of miR-1296-5 in DDP-resistant samples, and its expression had a negative correlation with circ_0017639 (Figure 3(e,f)). Similar results were noted in DDP-resistant cell lines (in contrast to their parental cells) and NSCLC cells (in contrast to HBE cells) (Figure 3(g)). The overexpression efficiency of circ_0017639 was confirmed by RT-qPCR, as displayed in Figure 3(h). Moreover, circ_0017639 inhibition elevated miR-1296-5p expression, but circ_0017639 overexpression had the opposing effect (Figure 3(i)). Together, circ_0017639 served as a miR-1296-5p sponge.

### Circ_0017639 regulated the resistance and malignancy of DDP-resistant NSCLC cells through sponging miR-1296-5p

**Figure 4.**
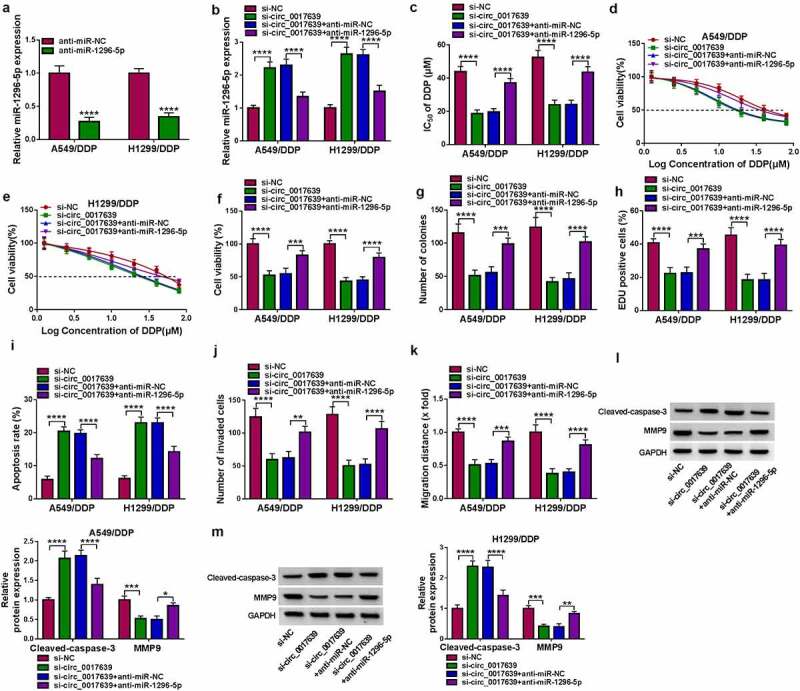
**Circ_0017639 sponged miR-1296-5p to regulate DDP-resistant NSCLC cell sensitivity and malignancy**. (a) Analysis of miR-1296-5p in DDP-resistant NSCLC cells transfected with anti-miR-NC or anti-miR-1296-5p by RT-qPCR. (b) Influence of miR-1296-5p inhibitor on miR-1296-5p in circ_0017639-inhibiting DDP-resistant NSCLC cells. (c and d) Effects of miR-1296-5p silencing on the IC_50_ value and viability of circ_0017639-inhibiting DDP-resistant NSCLC cells were assessed by MTT assay. (e-i) Impacts of miR-1296-5p knockdown on colony formation, proliferation, apoptosis, invasion, and migration of circ_0017639-inhibiting DDP-resistant NSCLC cells were analyzed by colony formation, EDU, flow cytometry, transwell invasion, and wound-healing assays. (j and k) Influence of miR-1296-5p silencing on MMP9 and Cleaved-caspase-3 protein levels in circ_0017639-inhibiting DDP-resistant NSCLC cells. *P < 0.05, **P < 0.01, ***P < 0.001, and ****P < 0.0001.

Given that circ_0017639 acted as a miR-1296-5p sponge, we further investigated whether circ_0017639 regulated DDP-resistant NSCLC cell resistance through binding to miR-1296-5p. Transfection with anti-miR-1296-5p caused an observable decrease in miR-1296-5p expression in DDP-resistant cells (Figure 4(a)). Moreover, the elevation of miR-1296-5p in circ_0017639-inhibiting DDP-resistant NSCLC cells was substantially restored after miR-1296-5p knockdown (Figure 4(b)). Silenced miR-1296-5p expression restored circ_0017639 knockdown-mediated decrease of the IC_50_ value and viability of DDP-resistant cells (Figure 4(c-d)). Furthermore, the inhibiting effect of circ_0017639 knockdown on DDP-resistant cell colony formation and proliferation was offset after anti-miR-1296-5p introduction (Figure 4(e-f)). As expected, miR-1296-5p inhibition counteracted the accelerating impact of circ_0017639 silencing on DDP-resistant cell apoptosis (Figure 4(g)). Additionally, decreased miR-1296-5p expression effectively abolished the inhibitory impact of circ_0017639 knockdown on DDP-resistant cell invasion and migration (Figure 4(h-i)). In addition, protein levels ofMMP9 and Cleaved-caspase-3 in DDP-resistant NSCLC cells mediated by circ_0017639 inhibition were restored after miR-1296-5p inhibitor introduction (Figure 4(j-k)). In sum, circ_0017639 sponged miR-1296-5p to regulate DDP-resistant NSCLC cell resistance.

### MiR-1296-5p directly targeted SIX1

**Figure 5.**
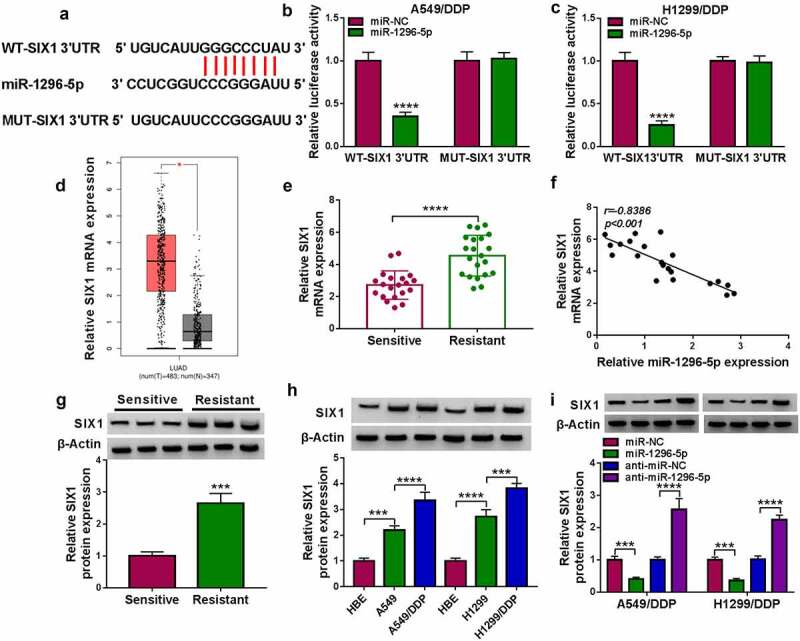
**SIX1 as a miR-1296-5p target was verified**. (a) Schematic illustrating the sequence alignment of SIX1 3’UTR with miR-1296-5p. (b and c) Dual-luciferase reporter assay assessed the luciferase activity of MUT-SIX1 3’UTR and MUT-SIX1 3’UTR plasmids in DDP-resistant NSCLC cells transfected with miR-1296-5p mimic or miR-NC. (d) The level of SIX1 mRNA in NSCLC through consulting the GEPIA database. (e) Analysis of SIX1 mRNA in 21 DDP-resistant NSCLC samples and 20 DDP-sensitive NSCLC samples by RT-qPCR. (f) Correlation of SIX1 mRNA and miR-1296-5p in DDP-resistant NSCLC samples was analyzed by Pearson’s correlation analysis. (g and h) Protein level of SIX1 in DDP-resistant NSCLC tissues, NSCLC cells, and DDP-resistant NSCLC cells was detected. (i) Impacts of miR-1296-5p mimic and inhibitor on SIX1 protein levels in DDP-resistant NSCLC cells. ***P < 0.001 and ****P < 0.0001.

To seek the downstream targets of miR-1296-5p, an online software targetScan was utilized. Among the many predicted targets, seven genes (SOX4 [43], ZEB2 [44], AVL9 [45], PTPN9 [46], RAB22A [47], ITGB8 [48] and SIX1 [49]) that have been reported to play an oncogenic role in lung cancer were further analyzed. In addition, miR-1296-5p overexpression significantly reduced the expression levels of ZEB2 and SIX1, especially SIX1 (Supplementary Fig. 1B). Figure 5(a) exhibits the potential binding between miR-1296-5p and SIX1. Moreover, miR-1296-5p mimic decreased the luciferase activity of the WT-SIX1 3’UTR plasmid, but there was no difference in the MUT-SIX1 3’UTR plasmid (Figure 5(b-c)). By consulting the gene expression profiling interactive analysis (GEPIA) database, we found that SIX1 was highly expressed in NSCLC patients (Figure 5(d)). Consistently, SIX1 was overexpressed in DDP-resistant samples (Figure 5(e)). Furthermore, the expression of SIX1 mRNA was negatively correlated with miR-1296-5p in DDP-resistant NSCLC samples (Figure 5(f)). We also observed a higher level of SIX1 protein in three DDP-resistant NSCLC samples than that in DDP-sensitive NSCLC samples (Figure 5(g)). In addition, higher levels of protein SIX1 protein were gained in NSCLC cells and DDP-resistant cells in contrast to their corresponding control cells (Figure 5(h)). As expected, miR-1296-5p overexpression decreased SIX1 protein levels in DDP-resistant cells, but miR-1296-5p inhibition had an opposing effect (Figure 5(i)). Overall, SIX1 acted as a miR-1296-5p target.

### MiR-1296-5p decreased DDP-resistant NSCLC cell resistance and malignancy via targeting SIX1

**Figure 6.**
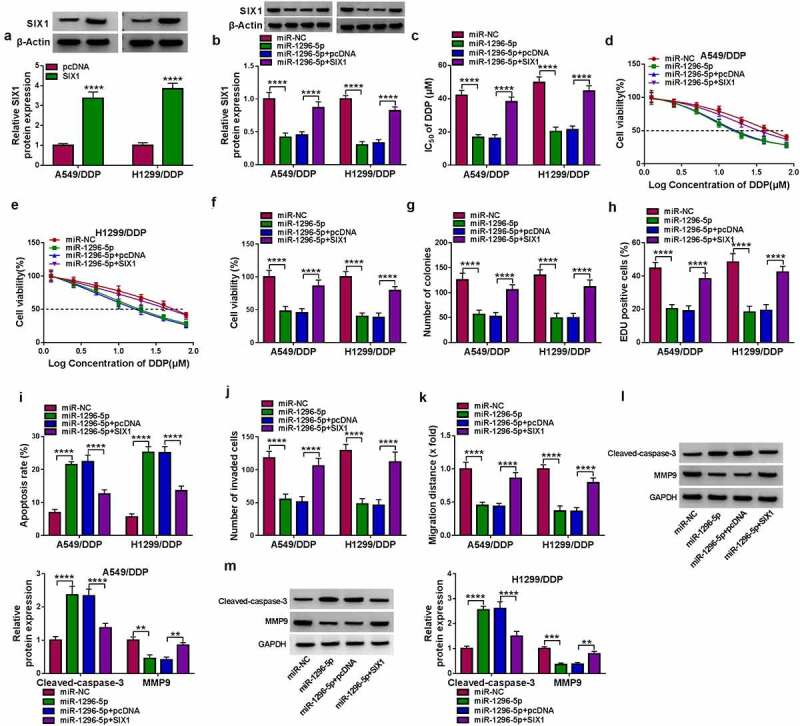
**MiR-1296-5p decreased DDP-resistant NSCLC cell resistance and malignancy via targeting SIX1**. (a) After SIX1 or pcDNA transfection, the SIX1 protein level was analyzed. (b) Influence of SIX1 introduction on the SIX1 protein level in miR-1296-5p-overexpressed DDP-resistant NSCLC cells was evaluated using western blotting. (c and d) Influence of SIX1 overexpression on the IC_50_ value and viability of miR-1296-5p-elevated DDP-resistant NSCLC cells was evaluated by MTT assay. (e-i) Impacts of SIX1 upregulation on colony formation, proliferation, apoptosis, invasion, and migration of miR-1296-5p-overexpressed DDP-resistant NSCLC cells were analyzed by colony formation, EDU, flow cytometry, transwell invasion, and wound-healing assays. (j and k) Effects of SIX1 upregulation on protein levels of MMP9 and Cleaved-caspase-3 in miR-1296-5p-overexpressed DDP-resistant NSCLC cells were evaluated by western blotting. **P < 0.01, ***P < 0.001, and ****P < 0.0001.

We then further addressed whether miR-1296-5p performs its function by targeting SIX1. Protein levels of SIX1 were prominently increased in DDP-resistant NSCLC cells after SIX1 transfection (Figure 6(a)). The decreased protein level of SIX1 induced by miR-1296-5p mimic was reversed by forcing the expression of SIX1 (Figure 6(b)). Moreover, elevated SIX1 expression abolished the decreased IC_50_ value and viability of DDP-resistant cells caused by miR-1296-5p overexpression (Figure 6(c-d)). Also, miR-1296-5p mimic repressed DDP-resistant cell colony formation and proliferation, and induced DDP-resistant cell apoptosis, but these trends induced by miR-1296-5p mimic were restored after SIX1 introduction (Figure 6(e-g)). In addition, miR-1296-5p elevation mediated repression on DDP-resistant cell migration and invasion was counteracted after SIX1 overexpression (Figure 6(h-i)). As expected, forced SIX1 expression overturned miR-1296-5p overexpression-mediated effects on protein levels of MMP9 and Cleaved-caspase-3 (Figure 6(j-k)). Collectively, miR-1296-5p targeted SIX1 to decrease the resistance and malignancy of DDP-resistant NSCLC cells.

### Circ_0017639 regulated SIX1 expression via adsorbing miR-1296-5p

**Figure 7.**
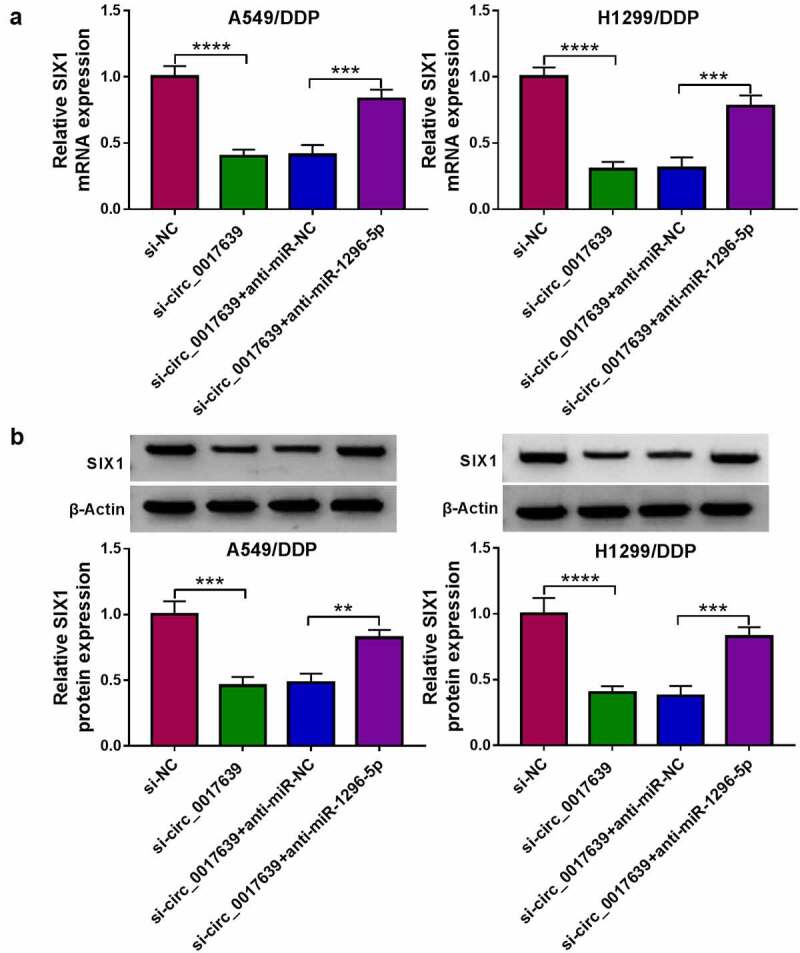
**Circ_0017639 regulated SIX1 expression via adsorbing miR-1296-5p**. (a and b) Analysis of SIX1 at mRNA and protein levels in DDP-resistant NSCLC cells transfected with si-NC, si-circ_0017639, si-circ_0017639+ anti-miR-NC, or si-circ_0017639+ anti-miR-1296-5p by RT-qPCR or western blotting. **P < 0.01, ***P < 0.001, and ****P < 0.0001.

Next, we further surveyed whether circ_0017639 regulates SIX1 expression via adsorbing miR-1296-5p. Circ_0017639 silencing reduced SIX1 at mRNA and protein levels, but this decrease was reversed after anti-miR-1296-5p introduction (Figure 7(a-b)). These data manifested that Circ_0017639 regulated SIX1 expression via sponging miR-1296-5p.

### *Circ_0017639 inhibition decreased xenograft tumor growth and sensitized cells to DDP* in vivo

**Figure 8.**
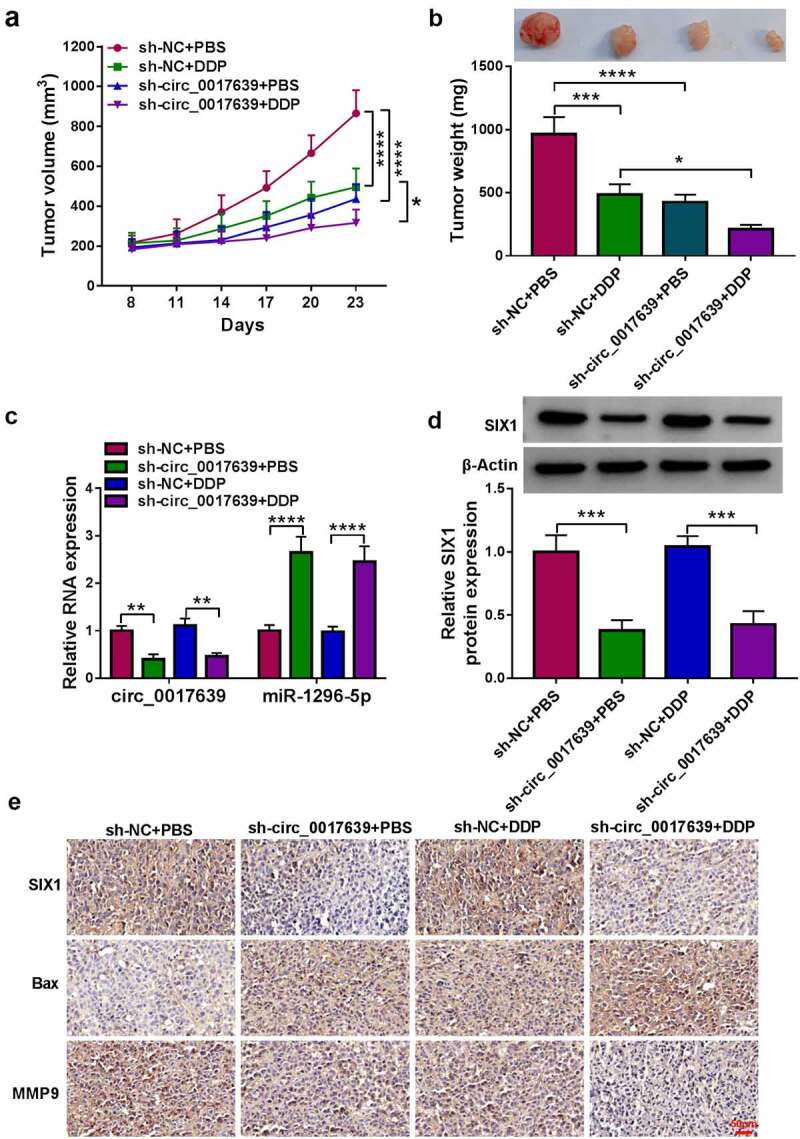
**Knockdown of circ_0017639 decreased xenograft tumor growth and sensitized cells to DDP *in vivo***. (a and b) The average tumor volume and weight of mice in sh-NC+PBS, sh-circ_0017639+ PBS, sh-NC+DDP, sh-circ_0017639+ DDP groups. (c and d) Analysis of circ_0017639, miR-1296-5p, and SIX1 protein in xenograft tumors in the above groups was done. (e) Analysis of SIX1 in xenograft tumors in the above groups by IHC. *P < 0.05, **P < 0.01, ***P < 0.001, and ****P < 0.0001.

To further support the above findings, we intraperitoneally administered DDP or PBS into nude mice injected with H1299/DDP cells carrying sh-circ_0017639 or sh-NC. Injection with H1299/DDP cells carrying sh-circ_0017639 decreased xenograft tumor growth and sensitized cells to DDP treatment (Figure 8(a-b)). Furthermore, the levels of circ_0017639 and SIX1 protein were decreased in sh-circ_0017639+ PBS and sh-circ_0017639+ DDP groups compared to their matched control group, but miR-1296-5p expression had an opposite tendency (Figure 8(c-d)). Also, the number of SIX1-positive cells was less in sh-circ_0017639+ PBS and sh-circ_0017639+ DDP groups with respect to their matched control group (Figure 8(e)). Together, circ_0017639 inhibition decreased xenograft tumor growth and sensitized cells to DDP *in vivo*.

## Discussion

Since DDP-induced resistance leads to treatment failure of NSCLC, it is essential to continue to explore the mechanism of DDP resistance in NSCLC. Our research described that circ_0017639 conferred DDP resistance through modulating the miR-1296-5p/SIX1 pathway in NSCLC.

CircRNAs have been demonstrated to exert essential roles in the response of cancer cells to DDP treatment [50]. Herein, we searched for a differentially expressed circRNA circ_0017639 in NSCLC samples from the GSE112214 dataset. The report of Li *et al*. exposed that circ_0017639 sponged miR-224-5p to elevate ubiquitin-specific protease 3 expression, resulting in elevating cell metastasis and proliferation in gastric cancer [13]. Nevertheless, circ_0017639 exerted an inhibiting impact on glioma cell metastasis and proliferation, which might be related to the tissue-specific expression [51]. A recent report showed that circ_0017639 was highly expressed in NSCLC samples and cell lines, and circ_0017639 overexpression promoted NSCLC cell proliferation, invasion, and migration via the PI3K/AKT signaling [52]. The difference between our study and the previous report [52] was to explore the function of circ_0017639 in DDP resistance in NSCLC. Our data showed higher levels of circ_0017639 in DDP-resistant samples and cell lines. Loss-of-function assays indicated that circ_0017639 silencing elevated DDP sensitivity of DDP-resistant NSCLC cells in xenograft models and facilitated DDP-resistant NSCLC cell apoptosis and decreased DDP-resistant NSCLC cell proliferation, invasion, and migration *in vitro*. These results suggested the contribution of circ_0017639 in NSCLC resistance to DDP.

Considering that circRNAs can function as miR sponges [35]. We observed that circ_0017639 was mainly distributed in the cytoplasm of DDP-resistant NSCLC cells through subcellular fractionation assay, implying that circ_0017639 might function as a miRNA molecular sponge. Furthermore, circ_0017639 as a miR-1296-5p sponge was identified through bioinformatics analysis and dual-luciferase reporter assay. Researchers had proved that miR-1296-5p could function as an inhibitor in gastric cancer by targeting cyclin-dependent kinase 6 and epidermal growth factor receptor [53], ERBB2-positive breast cancer by downregulating ERBB2 [17], in osteosarcoma through repressing notch receptor 2 expression [16]. A recent study described that miR-1296 repressed NSCLC cell invasion by blocking the Wnt signaling [54]. Our data exhibited that miR-1296-5p inhibitor could restore circ_0017639 silencing-mediated impact on DDP-resistant NSCLC cell resistance. Thus, we concluded that circ_0017639 regulated DDP resistance and tumor growth through sponging and repressing miR-1296-5p in NSCLC.

Subsequently, we discovered SIX1 as a miR-1296-5p target through bioinformatics analysis and dual-luciferase reporter assay. SIX1 could induce vascular endothelial growth factor-C expression, leading to cell migration in breast cancer [55]. It has been reported that cyclin A1 and cyclin D1 were transcriptional targets of SIX1 and that SIX1 promoted cell proliferation in breast cancer by increasing cyclin A1 expression [56] and in pancreatic cancer via increasing cyclin D1 expression [57]. Xia *et al*. revealed that the upregulation of miR-204 constrained NSCLC cell invasion and proliferation through targeting SIX1 [58]. Moreover, miR-186-5p decreased DDP resistance via downregulating SIX1 in NSCLC [49]. Also, FOXD2 adjacent opposite strand RNA 1 increased SIX1 expression by adsorbing miR-185-5p, thereby conferring DDP resistance of NSCLC [25]. Our data showed the upregulation of SIX1 in DDP-resistant NSCLC samples and cell lines, and SIX1 overexpression lessened the repressive effect of miR-1296-5p overexpression on DDP resistance in DDP-resistant NSCLC cells, manifesting that miR-1296-5p could regulate DDP resistance via targeting SIX1 in DDP-resistant NSCLC cells. Notably, circ_0017639 could regulate SIX1 expression via serving as a decoy of miR-1296-5p. Thus, we inferred that circ_0017639 regulated DDP resistance by regulating the miR-1296-5p/SIX1 pathway in NSCLC.

## Conclusion

Our findings highlighted the promoting function of circ_0017639 in DDP resistance of NSCLC. Moreover, circ_0017639 upregulation elevated SIX1 expression through adsorbing miR-1296-5p via acting as a miR-1296-5p molecular sponge, thereby conferring DDP resistance in NSCLC. The research provided the promoting effect of circ_0017639 on DDP resistance in NSCLC.

## Supplementary Material

Supplemental Material

## Data Availability

No new data were generated or analyzed in support of this research.

## Highlights:

1. Circ_0017639 was overexpressed in DDP-resistant NSCLC samples and cells.
2. Inhibition of circ_0017639 decreased DDP resistance of DDP-resistant NSCLC cells.
3. Circ_0017639 functioned as a miR-1296-5p molecular sponge.
4. SIX1 served as a miR-1296-5p target.
