# Supplementary figures and images for: Hsa_circ_0017639 regulates cisplatin resistance and tumor growth via acting as a miR-1296-5p molecular sponge and modulating sine oculis homeobox 1 expression in non-small cell lung cancer
Source: Bioengineered. 2022 Mar 28;13(4):8806–22. doi: 10.1080/21655979.2022.2053810 (PMC9161884; doi:10.1080/21655979.2022.2053810)

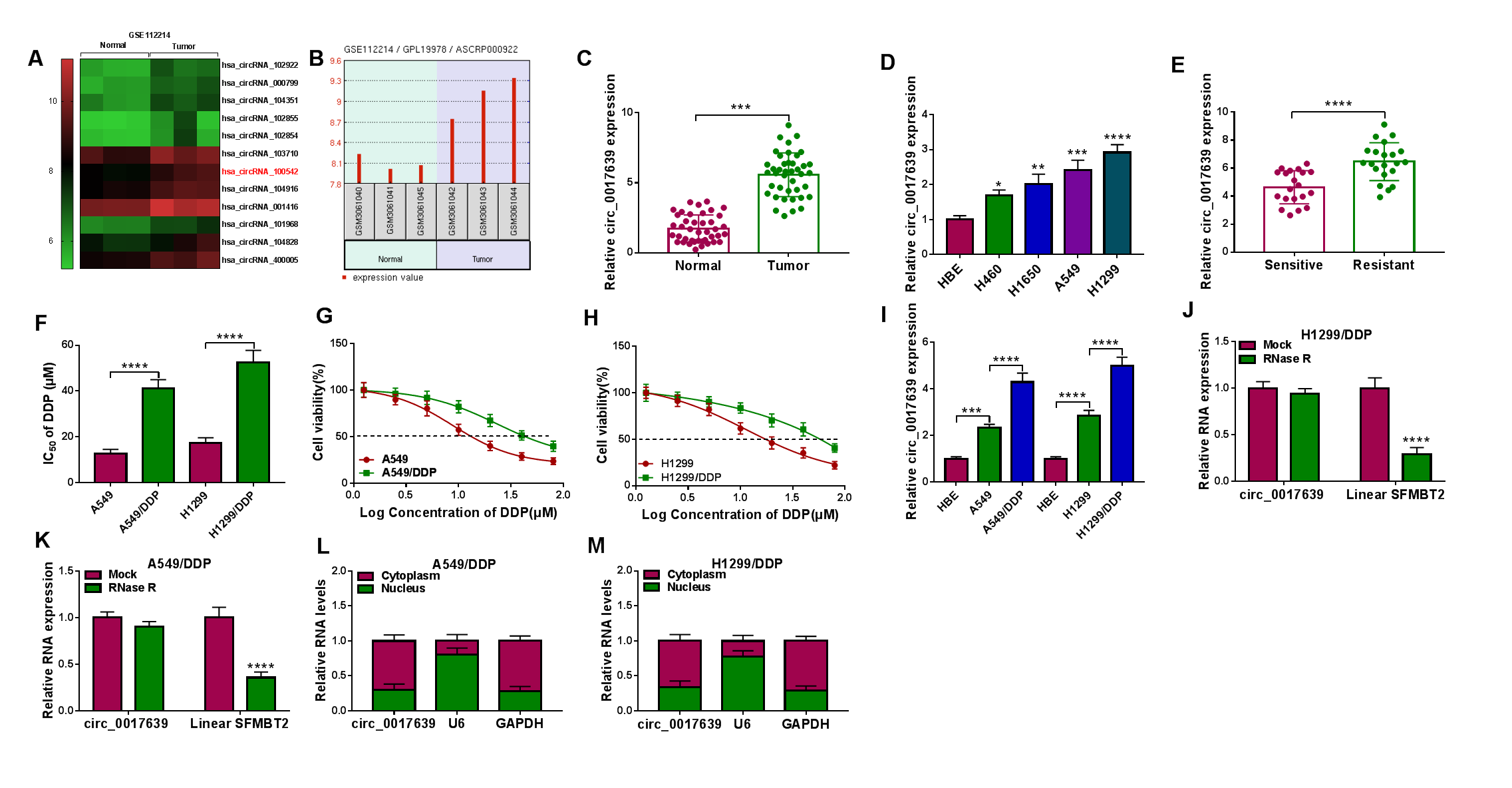

Supplement: Supplemental Material [file KBIE_A_2053810_SM9572.zip › supplementary Figure 1.tif]

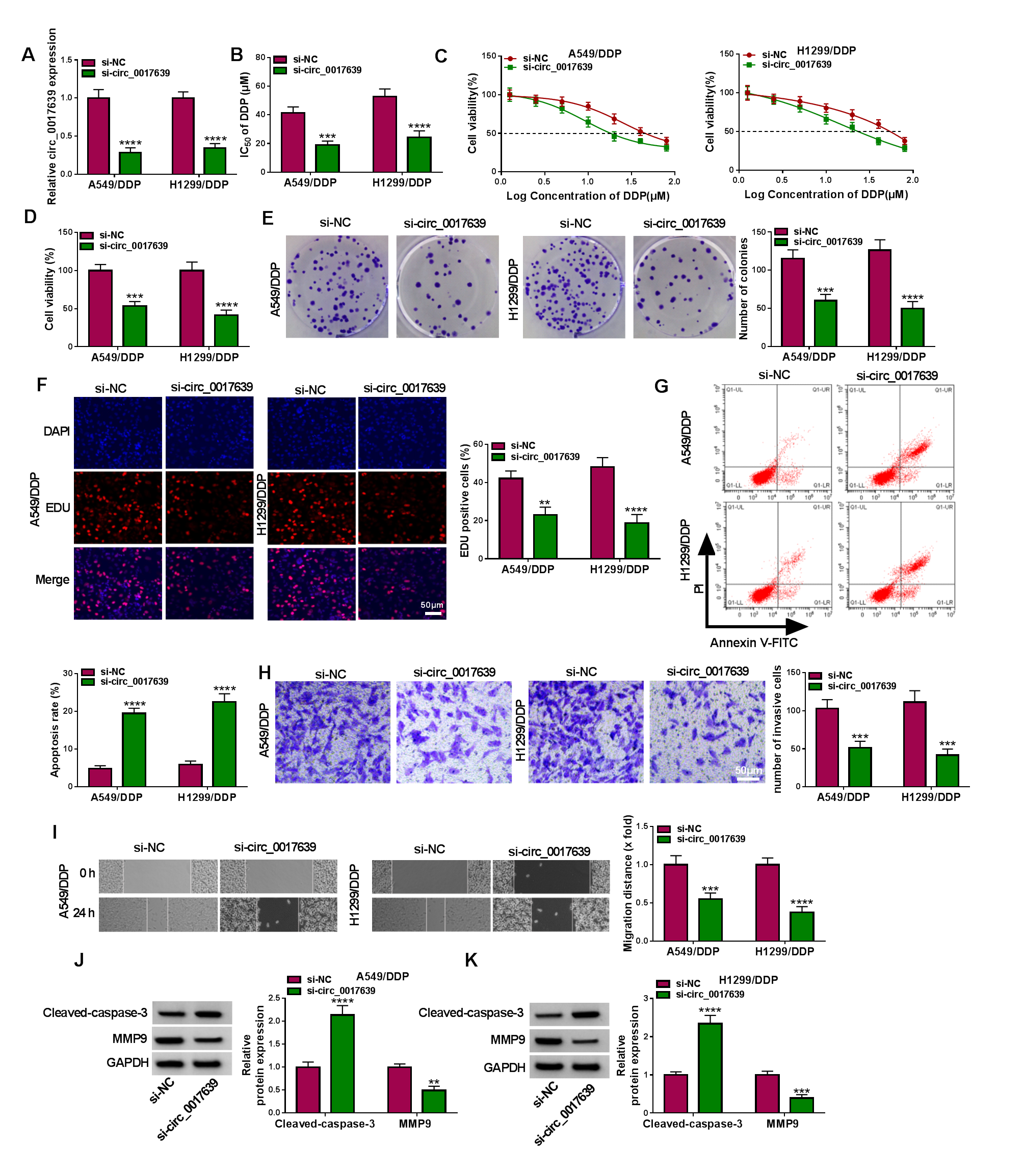

Supplement: Supplemental Material [file KBIE_A_2053810_SM9572.zip › supplementary Figure 2.tif]

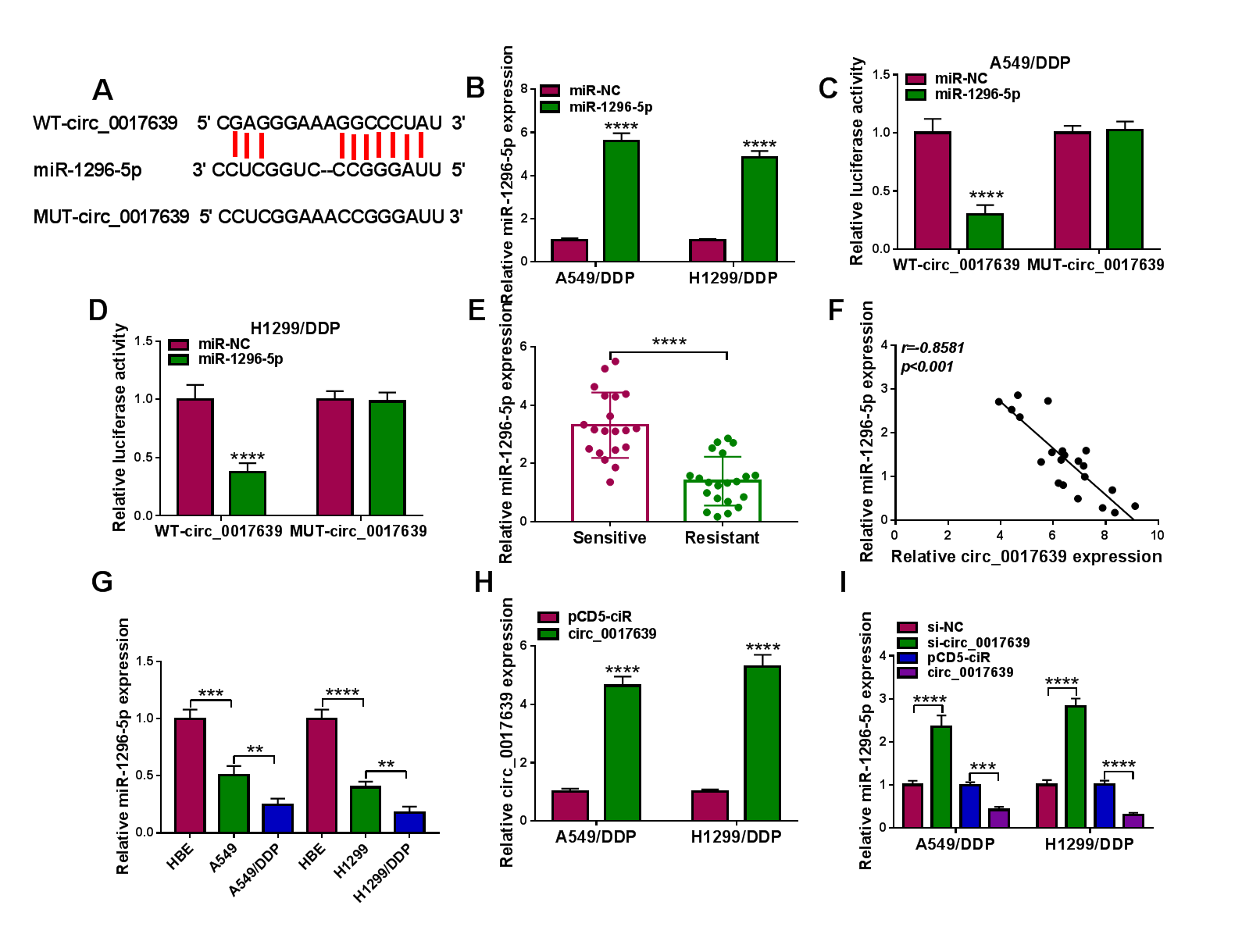

Supplement: Supplemental Material [file KBIE_A_2053810_SM9572.zip › supplementary Figure 3.tif]
